# *Plesiomonas shigelloides*: An Unusual Cause of Septic Abortion

**DOI:** 10.1155/2017/9363707

**Published:** 2017-05-28

**Authors:** Gilbert Cornut, Xavier Marchand-Senecal, Christiane Gaudreau, Jeremie Berdugo, Gilles Gariepy, Catherine Tremblay, Patrice Savard

**Affiliations:** ^1^Medical Microbiology and Infectious Disease, Centre Hospitalier de l'Université de Montréal-Hôpital Saint-Luc, Montreal, QC, Canada; ^2^Department of Microbiology, Infectiology and Immunology, Faculty of Medicine, Université de Montréal, Montreal, QC, Canada; ^3^Anatomical Pathology, Centre Hospitalier de l'Université de Montréal-Hôpital Saint-Luc, Montreal, QC, Canada; ^4^Department of Pathology and Cellular Biology, Faculty of Medicine, Université de Montréal, Montreal, QC, Canada; ^5^Obstetrics and Gynaecology, Centre Hospitalier de l'Université de Montréal-Hôpital Saint-Luc, Montreal, QC, Canada; ^6^Department of Obstetrics and Gynaecology, Faculty of Medicine, Université de Montréal, Montreal, QC, Canada

## Abstract

*Plesiomonas shigelloides*, the only oxidase-positive Enterobacteriaceae, is an inhabitant of freshwater and estuary ecosystems. We report the first possible case of* Plesiomonas shigelloides*-induced septic abortion. This 24-year-old female was successfully treated by dilatation and curettage as well as antimicrobial therapy.

## 1. Introduction

*Plesiomonas shigelloides* is a member of the family Enterobacteriaceae. It is mainly responsible for gastroenteritis but multiple extraintestinal infections have been reported such as septicemia and wound infections. Notable acquisition risk factors are consumption of raw or undercooked shellfish, foreign travel, and immunosuppression [1].

## 2. Case Presentation

A 11 weeks pregnant 24-year-old female sought medical attention for a 7-day progressive lower abdominal pain. She had no known medical condition with the exception of chlamydial cervicitis previously treated. She did not take any medications except for pregnancy related supplements and was an active smoker. Her current pregnancy was uneventful except for mild vaginal bleeding with a closed cervix assessed by ultrasound three weeks before presentation.

On admission, other than her abdominal pain, she denied bloating, diarrhea, or vomiting although close relatives who travelled with her did have diarrhea. The patient had recently travelled to the Caribbean and returned 7 days prior to presentation. During her trip she stayed in an all-inclusive resort. She denied any seafood consumption. She was found to be tachycardic and febrile at 39,3°C in the emergency room. Vaginal bleeding was initially similar to past bleeding episodes and did not orient clinicians toward an obstetrical infection. As such, Dengue and Zika virus serologies were ordered and returned negative in the next days. Beta-human chorionic gonadotropin level was 131,956 U/L on admission. A pelvic ultrasound revealed an intrauterine fetal pole with absent heart movement and a known stable anterior hematoma. Empirical piperacillin-tazobactam was administered with one dose of tobramycin and the patient spontaneously expelled product of conception and placenta in less than nine hours after triage in the emergency room. The patient progressed into a septic shock with transient vasopressors need; therefore she was brought to the operating room where dilatation and curettage was performed. Unfortunately, no material was found to be worth culturing at that time. Hemodynamic parameters improved promptly upon her return from the operating room. Four out of four blood culture bottles grew Gram-negative bacilli identified as* Plesiomonas shigelloides* by API 20NE (bioMérieux, Marcy L'Etoile, France) and confirmed by 16S rRNA sequencing. Antimicrobial susceptibility testing was performed by Kirby-Bauer following CLSI M02-A12 method and interpreted according to CLSI M100S26 recommendations [2, 3]. The bacterium was resistant to ampicillin with intermediate resistance to cefazolin and gentamicin and was found to be susceptible to ceftriaxone, ceftazidime, amoxicillin-clavulanate, piperacillin-tazobactam, ciprofloxacin, TMP-SMX, tobramycin, and ertapenem.* Chlamydia trachomatis* and* Neisseria gonorrhea* PCR on cervical swab were negative and, unfortunately, no bacterial cultures were realized. No stool specimen was obtained in the absence of GI symptoms. Neutrophil count peaked at 16.5 × 10^9^/L during hospitalization, creatinine remained within the normal limits, and liver function tests were slightly elevated. She was discharged from the intensive care unit after 24 hours and control blood cultures were negative after 48 hours of antimicrobial therapy. She was then switched to oral amoxicillin-clavulanate on day 3 of her admission and sent home on the fifth day to complete a 14-day course of antimicrobial therapy. On a follow-up visit five days after discharge, she was clinically well and her C-reactive protein was back to normal after reaching 256 mg/L on day two of her hospital stay.

**Figure 1:**
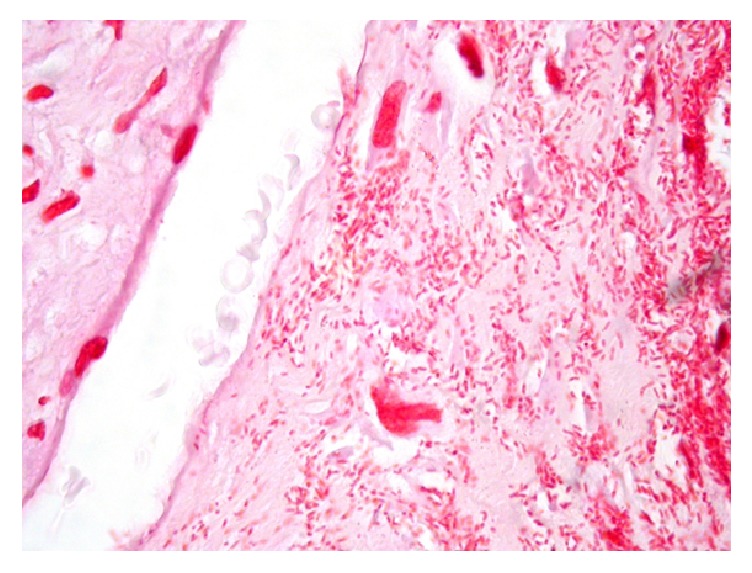
Gram-negative bacilli within chorionic villi (Gram stain, 1000x oil immersion).

Pathological analysis of the debris from the conceptus revealed chorioamnionitis, necrotizing deciduitis, and villitis. Gram stain on formalin fixed paraffin embedded tissue identified numerous Gram-negative bacilli within the chorionic villi and the placental membranes (Figure 1).

## 3. Discussion

Septic abortion is defined as an abortion complicated by fever and associated with endometritis and parametritis [4]. Risk factors include abortion with chemicals or instruments, pregnancy with an intrauterine device, and retained product of conception after either spontaneous or induced abortion. Endogenous vaginal flora is often involved and secondary bacteremia frequently ensues. Concordance between blood cultures and product of conception culture is high [5]. Spontaneous septic abortion is an uncommon event in developed countries with an associated mortality as high as induced septic abortion. It requires antimicrobial therapy combined with prompt surgical removal of conceptual debris [4]. Given the absence of risk factors in this case, the diagnosis was not considered until vaginal bleeding became significant, thus explaining delays in surgical intervention. Adequate workup for a suspected case of septic abortion should include both aerobic and anaerobic cultures of blood, cervical discharge, and products of conception [5]. Although no culture of gynecological origins was performed in this case, the septic abortion is most likely due to* Plesiomonas shigelloides* found in blood cultures since abundant Gram-negative rods were also identified within chorionic villi.

A pyosalpinx associated with* Plesiomonas shigelloides* was previously reported in an immunocompetent woman with presumed exposure from swimming in a contaminated sea bay [6]. Ascending infection through the vaginal canal was hypothesized as the acquisition mode. A dozen cases of neonatal sepsis have also been described [7]. As of today, there is no reported travel related septic abortion or any human case of* Plesiomonas shigelloides*-induced septic abortion but an animal case was previously described in a European otter [8]. Given our patient lack of gastrointestinal symptoms, ascending infection is also probable through either swimming in sea water, exposure to symptomatic close contacts, or asymptomatic gastrointestinal carriage.

*Plesiomonas shigelloides* is intrinsically resistant to penicillins due to beta-lactamase production and present variable susceptibility to aminoglycosides [1]. Septic abortion in a returning traveler with poor response to clindamycin and aminoglycosides, one of the first-line treatments, should raise the possibility of an atypical bacterial etiology.
